# Fecal Microbiota Transplantation in the Treatment Strategy of Inflammatory Bowel Disease: Molecular Mechanisms, Clinical Evidence, and the Transition Toward Defined Microbial Therapeutics

**DOI:** 10.3390/ijms272110001

**Published:** 2026-11-08

**Authors:** Anna Pető, David Sipos, Ágnes Kappéter, Zsofia Feiszt, Fanni Dembrovszky, Bernadett Halda-Kiss, Zoltán Péterfi

**Affiliations:** 1 Department of Medical Microbiology, Medical School, University of Pécs, 7623 Pécs, Hungary; anna.peto@aok.pte.hu; 2 1st Department of Internal Medicine, Medical School, University of Pécs, 7623 Pécs, Hungary; sipos.david@pte.hu (D.S.); kappeter.agnes@pte.hu (Á.K.); feiszt.zsofia@pte.hu (Z.F.); dembrovszky.fanni@pte.hu (F.D.); 3 Department of Infectology, Somogy County Kaposi Mór Teaching Hospital, 7400 Kaposvár, Hungary; kiss.bernadett2@pte.hu

**Keywords:** fecal microbiota transplantation, inflammatory bowel disease, ulcerative colitis, Crohn’s disease, gut microbiome, live biotherapeutic products, dysbiosis, short-chain fatty acids

## Abstract

Inflammatory Bowel Disease (IBD) comprises two clinically and pathologically distinct chronic intestinal inflammatory diseases, ulcerative colitis (UC) and Crohn’s disease (CD). Both diseases result from a dysregulated mucosal immune response to the commensal gut microbiota in genetically predisposed individuals. Because gut dysbiosis is a constant feature of both diseases, Fecal Microbiota Transplantation (FMT) has recently been investigated as a potential therapeutic strategy to restore a balanced microbiota composition in patients with IBD. There are five placebo-controlled randomized controlled trials (RCTs) in UC and several meta-analyses, which showed that treatment with FMT using material from multiple donors can lead to clinical and endoscopic remission in patients with mild-to-moderate UC and may thus double to triple the rate of remission compared with placebo (number needed to treat [NNT] ≈ 3–5). In contrast, evidence for CD is limited and inconsistent, with one promising pilot study being contrasted with a large multicenter induction trial that was stopped for futility. This review provides an update of the current molecular understanding of FMT-induced recovery of mucosal barrier function and inflammation control in intestinal inflammation (α-diversity restoration and subsequent donor strain engraftment, production of short-chain fatty acids and secondary bile acids, tryptophan–AhR signaling, IL-10-mediated induction of Tregs vs. suppression of Th17 cells and restoration of epithelial barrier function). The review also summarizes the current clinical evidence for the use of FMT in IBD, its safety, and the current regulatory status. It also describes recent developments in FMT, including the use of defined bacterial consortia, ‘live biotherapeutic products’ (LBPs), and donor–recipient pairing. Collectively, FMT provides evidence of principle for manipulating intestinal microbial ecosystems to treat immune-mediated intestinal inflammation.

## 1. Introduction

Inflammatory Bowel Disease (IBD) is a chronic, frequently relapsing and remitting disease of the gastrointestinal tract that involves the immune system. It is influenced by host genetic susceptibility and environmental factors, affects the epithelial barrier and is characterized by an aberrant mucosal immune response [1]. Although an increasingly broad armamentarium of biologic and small molecule therapies is available to treat IBD patients, including anti–tumor necrosis factor (anti–TNF) agents, integrins, IL–12/23p40, and Janus kinase (JAK) inhibitors, many patients do not achieve durable disease remission. Notably, most of these treatments do not directly address the microbial component of disease. The critical therapeutic gap in treating IBD has been further accentuated by consistent evidence of an altered gut microbiota in IBD. The most promising approach to fill this treatment gap is FMT (Fecal Microbiota Transplantation), which has entered clinical practice as a therapeutic option for recurrent *Clostridioides difficile* infection (rCDI) [2,3].

Initially, the idea of transferring normal gut microbes to a diseased gut was inspired by the tremendous success of FMT in treating rCDI, with cure rates of up to 90%. In line with the “dysbiosis paradigm,” FMT has also been proposed to treat other chronic infectious or inflammatory diseases, based on the concept of an inverse correlation between disease activity and the level of dysbiosis. However, IBD presents a different ecological challenge than rCDI. In the chronically diseased gut, the resident gut microbiota is not acutely suppressed by environmental factors but is chronically maladapted to grow in the inflamed gut mucosa with high oxygen and nitrate concentrations. In contrast to acute insults in rCDI, the diseased gut microbiota is therefore difficult to remove and replace with donor microbes [2,4].

This review focuses on adult Crohn’s disease and ulcerative colitis. 

## 2. The Microbial Background: Dysbiosis in IBD

In the gut microbiome, α-diversity describes microbial diversity within a single sample (stool or mucosa), and β-diversity describes microbial diversity between samples. Reduced α-diversity is a common feature of disease-associated dysbiosis. Studies have shown that IBD is characterized by a reduction in α-diversity within the phylum *Firmicutes* and decreased temporal stability of dominant taxa. Instead, there is an increased abundance of facultative anaerobic bacteria within the phylum *Proteobacteria*, particularly within the family *Enterobacteriaceae*. Several large multi-cohort metagenomic analyses have described depletion of beneficial obligate anaerobes including *Faecalibacterium prausnitzii*, *Roseburia* spp., *Eubacterium* rectale and *Ruminococcus* spp. Conversely, pathogens such as *Ruminococcus gnavus*, *Fusobacterium* spp., *Enterococcus* spp., adherent-invasive *Escherichia coli* (AIEC), and *Campylobacter* spp. are more abundant [5,6]. The extent of dysbiosis is generally greater in CD than in UC and is typically greater in mucosal samples than in feces [7].

Several studies have described differences in microbial abundance between UC and CD (Table 1).

In UC, the typically depleted taxa include *Eubacterium rectale*, *Akkermansia mucinophyla*, *Faecalibacterium prausnitzii*, *Alistipes shahii*, *Blautia longum*, and the genera *Coprococcus, Dialister,* and *Lachnospira*. In contrast, the abundance of *Escherichia coli*, *Shigella* spp., *Bacteroides* spp., *Ruminococcus gnavus*, *Bifidobacterium* spp., *Enterococcus* spp. (*E. faecalis*), and *Streptococcus salivarius* correlates with disease activity in UC [5,6,9,10]. 

In CD, dysbiosis is more pronounced than in UC. Studies have shown that *Faecalibacterium prausnitzii*, *Roseburia* (including *R. intestinalis* and *R. hominis*), *Bacteroides* spp. and *Prevotella* spp., *Clostridium* clusters XIVa and IV, *Christensenellaceae*, *Coriobacteriaceae*, *Bifidobacterium*, *Akkermansia muciniphila*, *Ruminococcus bromii*, and *Agathobacter*, *Romboutsia*, and *Butyricicoccus* are depleted in CD, while *Escherichia coli*, *Klebsiella pneumoniae*, *Veillonella* spp., *Fusobacterium* spp., *Actinomyces* spp., *Ruminococcus gnavus*, and *Actinobacteria*/*Gammaproteobacteria* are enriched in patients with CD. CD is characterized by enrichment of an enrichment of *E. coli* and ESKAPE pathogens, including *Klebsiella pneumoniae*, *Staphylococcus aureus*, *Acinetobacter baumannii*, and *Pseudomonas aeruginosa* [5,10,11,12]. 

IBD is characterized by intermittent periods of dysbiotic gut microbiota in addition to chronic maladaptive microbiota. Work from the Integrative Human Microbiome Project (iHMP/HMP2) found that active disease in UC and CD was associated with periods of dysbiotic the gut microbiota characterized by decreased alpha diversity of the fecal microbiota with decreased representation of the obligate anaerobes, particularly butyrate-producing *Firmicutes* [8]. Recent studies have characterized the IBD gut virome, which shows increased richness of *Caudovirales* bacteriophages and decreased overall viral diversity [13,14]. Furthermore, recent studies have identified an altered gut mycobiome in IBD patients, usually characterized by decreased biodiversity and decreased representation of beneficial species such as *Saccharomyces cerevisiae*, with the harmful pathogenic yeast *Candida albicans* representing the most increased genus in UC and CD patients (Table 2). This finding is not universal and should not be interpreted as a disease-specific signature. [9,15,16]. 

It is unclear whether dysbiosis is a cause or consequence of the inflamed gut, but it is thought to be a self-reinforcing “circular causality” between altered gut microbiota, decreased production of protective metabolites by gut bacteria, and the inflamed gut [17]. Importantly, the observed changes in gut microbiota in IBD support the therapeutic concept of FMT to restore a “normal” or “healthy” diverse gut microbiota containing many obligate anaerobes.

**Table 2:** Comparative mycobiomal and viromal alterations in Ulcerative Colitis (UC) and Crohn’s Disease (CD). Reported mycobiome alterations represent cohort-level associations rather than consistent diagnostic features of UC or CD. Findings may differ according to disease activity, intestinal location, treatment exposure, sample type, sequencing platform, and bioinformatic analysis. Abbreviations: IBD—Inflammatory Bowel Disease, ASCA—Anti-*Saccharomyces cerevisiae* antibodies.

Feature	Ulcerative Colitis	Crohn’s Disease	References
Overall mycobiome disturbance	Less consistent; milder	More pronounced; increased fungal diversity	[7,18]
*Basidiomycota*/*Ascomycota* ratio	Increased (shared IBD signature)	Increased (shared IBD signature)	[19]
*Candida albicans*	Increased; high damage candidalysin strains identified	Increased (more consistent); + *C. parapsilosis*	[6]
*Candida tropicalis*	Variable	Elevated (in active disease)	[6]
*Saccharomyces cerevisiae*	Decreased	Variable, with both depletion and enrichment reported; ASCA strongly CD-associated	[9,20]
Other distinctive fungi	*Saccharomyces paradoxus*, *S. kudriavzevii* enriched; *Exophiala mesophila* depleted	*Malassezia restricta*, *Debaryomyces hansenii* enriched	[9]
Viral α-diversity	Reduced (lowest)	Reduced	[9]
Bacteriophage shift	*Caudovirales* + *Escherichia*/*Enterobacteriaceae* phages enriched (mucosa)	Bacteriophage depletion (ileal); phages vs. *Staphylococcus*/*Faecalibacterium*/*Escherichia*	[21]
Eukaryotic viruses	Less prominent	Elevated, esp. Torque teno virus (correlates with pathobionts)	[21]
Phage lifecycle	Shift to temperate/lysogenic; loss of transkingdom correlations	Shift to temperate/lysogenic; disrupted phage–bacteria mutualism persisting in remission	[9]
*Microviridae*	Reduced (variable; some UC mucosa increased)	Reduced	[21]

## 3. Fecal Microbiota Transplantation and Its Molecular Mechanisms

The mechanism(s) by which FMT exerts therapeutic effects in IBD are not fully established and are believed to involve more than the simple eradication of single pathogenic microorganisms. Rather, FMT’s therapeutic effects are associated with changes in the microbial ecosystem and/or its function. Several putative mechanisms of action have been proposed and tested in experimental models and in clinical trials, including mechanistic sub-studies. The following mechanisms should therefore be regarded as biologically plausible and variably supported by human FMT studies, mechanistic substudies, and experimental models.

### 3.1. Restoration of Bacterial Diversity and Donor Engraftment

Increased α-diversity following FMT has been associated with favorable clinical outcomes in UC, although its direct contribution to therapeutic efficacy remains uncertain. A review of the features of the microbiota in clinical trials of UC FMT found that higher donor and recipient α-diversity and a sustained shift from a low-diversity, *Bacteroides*-dominated fecal microbiota toward a butyrate-producing profile correlated with clinical response [22]. These findings support a potential role for microbial ecosystem restoration but do not establish that increased α-diversity itself is a necessary or sufficient mediator of remission. The TURN and FOCUS trials evaluated FMT in active UC and reported differing primary outcome results. In TURN, donor FMT did not significantly improve the composite of clinical remission and endoscopic response at week 12 compared with autologous FMT (30.4% [7/23] vs. 20.0% [5/25]; *p* = 0.51; based on an intention-to-treat analysis [23]. In contrast, FOCUS demonstrated a significant benefit of intensive multidonor FMT over placebo for steroid-free clinical remission with endoscopic remission or response at week 8 (27% [11/41] vs. 8% [3/40]; risk ratio 3.6, 95% CI 1.1–11.9; *p* = 0.021; based on a modified intention-to-treat analysis [24]. In both trials, an increase in diversity was observed in both responders and non-responders in the short term (up to 8–16 weeks after FMT) but not in the latter in the long term [22]. 

The amount of donor strain that engrafts into a patient’s gut can vary greatly by strain and between patients, influencing clinical outcomes. Analysis of the data from the Sokol trial demonstrated that higher levels of donor engraftment (or donor strains) correlated with lower rates of relapse in Crohn’s disease and that 38% of the changes to a patient’s gut microbiota caused by FMT were due to the replacement of a patient’s native strains with equivalent donor strains not detected by 16S rRNA gene-based profiling of stool [25]. These findings support a possible relationship between engraftment and clinical response, but they do not show that engraftment causally drives remission.

Others have found that the properties of the recipient microbiome, in addition to those of the donor, are critical determinants of donor engraftment and that certain “gatekeeper” species of the recipient’s microbiome can influence the engraftment of an FMT donor [26,27]. Strain-specific outcomes are generally predictable a priori, but such prediction has not yet been validated for routine clinical decision-making [28]. Medications that reduce diversity and decrease inflammation (such as reduced post-antibiotic calprotectin) prior to FMT can facilitate engraftment of infused donor microbes; however, their effect on sustained clinical benefit remains uncertain.

### 3.2. Short-Chain Fatty Acids

Short-chain fatty acids (SCFAs), produced by microbial degradation of dietary components, are important effector molecules. Decreased production of fecal butyrate has been noted in active IBD and restored in responders to FMT [20,29]. A key feature of the FMT donor microbial community associated with active IBD is a stable subset of butyrate-producing microbes (e.g., *Eubacterium hallii*, *Roseburia inulinivorans*) that enablesrestored colonization by potential responders to form SCFAs and secondary bile acids [29]. These observations suggest an association between SCFA-related functions and clinical response, but do not establish that restoring butyrate production is required for therapeutic efficacy. Butyrate, one of the most extensively studied SCFAs, serves as an important energy source for colonocytes and can modulate gene expression by inhibiting histone deacetylases. It can promote the extrathymic differentiation of naïve CD4+ T cells into regulatory T cells [30].

SCFAs and their corresponding receptors also play a key role in the immune system, activating G-protein-coupled receptors (GPRs) on dendritic cells and macrophages to suppress inflammatory cytokine production. GPR43 and GPR109A are specifically activated by acetate and butyrate [13,17]. Experimental and computational studies have implicated free fatty acid receptors, including FFAR2 and FFAR4, in regulating intestinal inflammation, although their individual contributions to the therapeutic effects of FMT remain uncertain [31,32].

### 3.3. Bile Acid Metabolism

Secondary bile acids are other products made by microbes in the gut lumen. The liver produces primary bile acids, but in the gut microbes convert them to secondary bile acids via bile salt hydrolases (BSH) and 7α-dehydroxylating bacteria. Secondary bile acids, lithocholic acid (LCA) and deoxycholic acid (DCA), can act as anti-inflammatory signals. They activate the membrane receptor TGR5, which promotes intestinal barrier function, and Treg induction, and the nuclear receptor FXR, which inhibits NF-κB-mediated pro-inflammatory gene expression [33,34]. FMT has been found to restore these secondary bile acid pathways in responders to FMT and to improve the condition of patients with IBD with bile acid malabsorption [29,35]. Nevertheless, these clinical observations remain correlative. 

### 3.4. Tryptophan–AhR Signaling, Immune Modulation, and Barrier Repair

The microbial tryptophan catabolites (indoles) act on the aryl hydrocarbon receptor (AhR) and induce production of IL-22, antimicrobial peptides, and other molecules that support the intestinal epithelial barrier. The activity of this AhR-activating pathway is decreased in IBD patients and can be restored in individuals with IBD who receive FMT [17,36,37]. The causal contribution of AhR activation to clinical remission has not been established in human FMT trials. Experimental studies suggest that FMT can modulate intestinal immune responses by increasing the number of IL-10-producing mucosal regulatory T cells, reducing proinflammatory cytokine production by Th17 cells, and inhibiting activation of the NF-κB signaling pathway [38]. IBD pathology is associated with dysregulated immune responses, including an imbalance between helper T-cell subsets that produce proinflammatory vs. anti-inflammatory cytokines, including TNF- vs. IL-10 [38]. However, immunological changes have been characterized less consistently in human IBD FMT studies than in experimental models. Furthermore, the FMT-induced gene expression signature in intestinal mucosa includes genes that restore normal intestinal architecture, most notably by upregulating genes that encode components of tight junctions between epithelial cells as well as genes involved in calcium signaling. Notably, two genes, GBP5 and IRF4, were upregulated.

FMT responders and associated with clinical remission [39]. *F. prausnitzii*, whose depletion is most persistent in IBD, is a keystone anti-inflammatory bacterium that produces butyrate, excretes an NF-κB-inhibitory peptide, and induces IL-10 production; however, the restoration of *F. prausnitzii* alone is not sufficient to induce remission [40,41].

### 3.5. Virome, Mycobiome Modifications

FMT influences the recipient phageome to resemble that of the donor(s), and in particular, a phage (kiOS01_B), contained within the *Oscillospiraceae*-host genome, correlates with clinical remission in UC in both the FOCUS and LOTUS trials [42]. Fungal dynamics in FMT vary by disease. In CDI, the presence of *Candida albicans* was linked to reduced FMT efficacy; however, in UC patients, high pre-FMT Candida abundance was associated with clinical benefit [43]. Notably, FMT in CD patients led to a decrease in the abundance of opportunistic pathogenic fungi and an increased abundance of antifungal immunity [15]. These observations remain exploratory, and prospective human studies are needed to confirm the mycobiome’s or virome’s independent contribution to the efficacy and safety of FMT (Table 3).

**Table 3:** Molecular mechanisms and clinical implications of fecal microbiota transplantation in ulcerative colitis and Crohn’s disease. Abbreviations: AMP—antimicrobial peptide; BA—bile acid; CD—Crohn’s disease; DCA—deoxycholic acid; FMT—fecal microbiota transplantation; FXR—farnesoid X receptor; IL—interleukin; LCA—lithocholic acid; NF-κB—nuclear factor kappa B, SCFA—short-chain fatty acid, TGR5—Takeda G protein-coupled bile acid receptor 1; TGF-β—transforming growth factor beta; Treg—regulatory T cell; UC—ulcerative colitis; UDCA—ursodeoxycholic acid; ZO-1—zonula occludens-1, ↓ means decreased abundance and ↑ means increased abundance.

Molecular Pathway/Component	Mechanism in UC Post-FMT	Mechanism in CD Post-FMT	Clinical/Efficacy Implications	References
SCFA/butyrate → Treg + barrier	Engraftment of SCFA producers (*Oscillospiraceae*, *Christensenellaceae*, *Lachnospiraceae*); ↑butyrate/acetate, ↓NF-κB p65, ↑Claudin-1/Occludin/ZO-1; response tracks luminal butyrate	Restoration of butyrogenic taxa expected but less consistently demonstrated; deeper baseline butyrate deficit harder to reverse	UC: ~30% remission, best with colonic route + repeat dosing; butyrate a candidate response biomarker. CD: mechanistic support mostly preclinical	[20,44]
Secondary bile acids (LCA/DCA/UDCA) → TGR5/FXR	Restoration of 3-oxo-LCA associated with FMT success; corrects bile acid malabsorption (↓C4); TGR5-mediated barrier gain	FXR expression reduced in inflamed CD colon; secondary BA depletion during flares; FXR agonism protective in models	Bile acid shift is a determinant of FMT response in UC; FXR/TGR5 axis a rational adjunct target in both	[33,34]
Immune shift (Th1/Th17→Treg/IL-10/TGF-β)	↑mucosal Tregs, ↓Th17 and CD8+, ↓IFN-γ; transcriptomic downregulation of proinflammatory defense pathways in responders	↑mucosal Tregs without consistent Th17 change; IL-10-dependent APC reprogramming	Anti-inflammatory shift only in engrafted responders; non-responders show no transcriptomic change	[20,38,39,44]
Mucus (MUC2) + antimicrobial peptides	Mucus layer/goblet cell restoration; ↑Muc1/Muc4, cathelicidin, S100A8; ↑tight-junction/calcium-signaling genes in responders	Restoration of Muc2, defensins (Defa), AhR-linked antimicrobial programs shown in humanized models	Barrier/AMP repair requires coordinated host response; predicts durability of remission	[34,38,45]
Overall clinical evidence	Multiple RCTs; induction of remission in a subset; guidelines restrict to trials	Limited, largely uncontrolled cohorts; efficacy unclear	FMT investigational in both; stronger rationale and data in UC	[7,44]

## 4. Clinical Evidence in Ulcerative Colitis

### 4.1. Induction of Remission

Five placebo-controlled RCTs constitute the core evidence base for FMT in mild-to-moderate UC. Their designs vary substantially in delivery route, donor pooling, dosing intensity, and antibiotic preconditioning, complicating cross-trial comparison but also highlighting which variables matter (Table 4).

In UC trials, 4 out of 5 reached their primary endpoint. The Moayyedi (*N* = 38 UC patients, *N* = 37 placebo control), FOCUS (*N* = 42 UC, *N*. = 43 control), Costello (*N* = 38 UC, *N* = 35 control), and LOTUS (*N* = 15 UC, *N* = 20 control) trials demonstrated significantly higher remission rates in the FMT groups compared with controls, in contrast the TURN trial (*N* = 23 FMT, *N* = 25 control) reported a numerically higher rate of achieving its composite primary endpoint without a statistically significant difference between groups. Across these studies, the reported primary endpoint rates in the FMT groups ranged from approximately 24% to 53%. Importantly, the 40-infusion FOCUS protocol achieved remission rates comparable to the far less intensive three-infusion Costello protocol (27% vs. 32%) [24,46]. 

Remission rates in the FOCUS and Costello trials were similar, but these studies had too few participants to rule out the need for more intensive administration than was used. The two studies differed in several parameters of the fecal donors and FMT preparations, as well as the routes of administration, concomitant treatments, and study end points. Therefore, the optimal dose, frequency, route of administration, and treatment duration for FMT remain unknown. Notably, the first combination trial, LOTUS, which used oral, lyophilized donor feces as FMT in combination with antibiotic preconditioning, achieved the highest remission rate so far in a clinical trial (53%), in a small trial that was terminated prematurely due to the SARS-CoV-2-pandemic [47]. Importantly, in the Moayyedi trial (75 randomized patients, 9/38 and 2/37 responders), 7/9 responders received stool from a single donor [48].

**Table 4:** Landmark randomized controlled trials of fecal microbiota transplantation for induction of remission in ulcerative colitis. Note: The number of randomized patients is presented separately from the denominators used for the primary endpoint analyses. Data are reported as n/N (%), where n is the number of patients achieving the primary endpoint, and N is the number of patients included in the corresponding analysis. In the FOCUS trial, 85 patients were randomized, but 81 were included in the modified intention-to-treat analysis. Primary endpoint definitions and assessment time points differed across trials. The TURN trial did not demonstrate a statistically significant treatment benefit.CI—confidence interval; NS—not significant; OR—odds ratio; RD—risk difference; RR—risk ratio.

Trial	Randomized Patients (FMT/Control)	Route	Donors	Primary Endpoint Achieved, n/N (%) (FMT vs. Control)	Effect Estimate
Moayyedi et al. (2015) [48]	75 (38/37)	Weekly enema × 6 weeks	Multiple donors, administered individually	9/38 (24%) vs. 2/37 (5%)	RD 17% (95% CI 2–33)
Rossen et al. (2015, TURN) [23]	48 (23/25)	Nasoduodenal × 2	Patient-directed	7/23 (30%) vs. 5/25 (20%)	*p* = 0.51; NS
Paramsothy et al. (2017, FOCUS) [24]	85 (42/43)	Colonoscopy + enema 5×/weeks × 8 weeks	Pooled (3–7)	11/41 (27%) vs. 3/40 (8%)	RR 3.6 (95% CI 1.1–11.9)
Costello et al. (2019) [46]	73 (38/35)	Colonoscopy + 2 enemas over 1 week	Pooled (3–4), anaerobic	12/38 (32%) vs. 3/35 (9%)	OR 5.0 (95% CI 1.2–20.1)
Haifer et al. (2022, LOTUS) [47]	35 (15/20)	Oral lyophilized capsules × 8 weeks + antibiotics	Single donor	8/15 (53%) vs. 3/20 (15%)	OR 5.0 (95% CI 1.8–14.1)

### 4.2. Meta-Analytic Estimates

Overall, meta-analyses supported the use of FMT for IBD to improve clinical, endoscopic, and histological remission. The pooled risk ratio for induction of clinical remission was 1.88 (1.37 to 2.57) in the 2023 Cochrane Review, and the pooled risk ratio for the combination of clinical and endoscopic remission was 2.13 (1.51 to 3.02), with zero percent variability explained by differences between studies [49]. The number needed to treat was found to be approximately 5 based on a 28% (vs. 9%) clinical remission rate in the 4 RCTs included in the 2017 pooled analysis [50]. A subsequent analysis restricted to the double-blinded RCTs had an overall Odds Ratio of approximately 4.00 for the combination of clinical and endoscopic remission and, importantly, showed no difference between single donors and pooled donors, between fresh and frozen preparations, between delivery routes, and between dosing frequencies [51]. The most consistent benefit for induction of combined clinical and endoscopic remission was found for the colonoscopy-plus-enema approach in an updated 2026 meta-analysis [52]. Cochrane rated the evidence for all induction endpoints as low certainty, mainly because of high risk of bias and imprecision. The RCTs included in the meta-analyses had fewer than 600 participants.

### 4.3. Maintenance and Durability

Evidence supporting maintenance FMT is limited and of low certainty. In the Sood pilot study, patients received FMT by colonoscopy every 8 weeks and maintained clinical remission in 87% of patients compared with 67% receiving placebo (*p* = 0.111), with significantly improved endoscopic and histologic remission [53]. In LOTUS maintenance, all 4 patients who continued to receive oral capsules of lyophilized FMT stool remained in remission at week 56, as compared to 0/4 patients who discontinued FMT [47]. Finally, the FMT-AID study found that combining multi-donor FMT with an anti-inflammatory diet significantly enhanced both induction and 48-week maintenance of deep clinical, endoscopic, and histologic remission [38,54].

## 5. Clinical Evidence in Crohn’s Disease

The available clinical evidence discussed here includes two randomized controlled trials and one open-label, uncontrolled study in adult patients with CD. The studies were small, heterogeneous, and evaluated different clinical, endoscopic, and microbiological outcomes. Therefore, the results of the exploratory and uncontrolled studies differ from the negative results of the only multicenter randomized trial on CD and therefore must be interpreted differently from the latter studies. Sokol and co-workers reported a pilot single-blind sham-controlled study in 17 patients with colonic or ileocolonic CD in steroid-induced remission. Although the primary endpoint of engraftment was not met, several other endpoints, including steroid-free clinical remission at week 10, were favorably affected by FMT (87.5% vs. 44.4%, *p* = non-significant), as well as CDEIS scores, which declined significantly in FMT-treated patients but not in sham-treated patients. Because of the small number of patients in this exploratory trial, the secondary endpoints must be considered with caution and thus not be transferred to other patient cohorts. The degree of engraftment correlated with the degree of clinical response and conversely with the degree of clinical flare in patients who did not engraft [55]. 

The most recent RCT was a multicenter, double-blind, placebo-controlled induction study in 15 CD patients which was terminated early for futility as no patient in the FMT group achieved the combined clinical and endoscopic outcome at week 8 (0% vs. 8.3% in placebo-treated patients). FMT did not shift the microbiota in patients who received it [56]. These results contrast with the favourable outcomes reported in earlier pilot studies and do not provide evidence supporting the efficacy of FMT for inducing remission in patients with CD. However, the small sample size and early termination limit the conclusions that can be drawn.

Yang and co-workers compared several methods of delivery in an open-label, uncontrolled study in 10 patients with active CD [57]. In this study, it was not possible to determine treatment effectiveness because it was not randomized and used no comparator.

An additional meta-analysis of CD FMT trials reported clinically apparent improvements in patients’ symptoms, but the pooled data were based mostly on uncontrolled cohorts (the number of RCTs conducted on CD is extremely small), so there is a high risk of bias. Most reported clinical remission in about 50–57% of patients treated with FMT, with a reduction in the CDAI score in responders [58]. No meta-analytic evaluation of evidence for maintenance of FMT-induced remission in CD was found. 

The less consistent responses to FMT in CD compared to UC can be ascribed to several factors, i.e., resistance of the resident microbiota to colonization; transmural vs. mucosal inflammation; host genes (e.g., NOD2, ATG16L1, CARD9) that are impaired in handling FMT microbes by the innate immune system; high clinical heterogeneity; and the frequent involvement of the small bowel, not amenable to FMT by colonic delivery [2,40]. Studies on FMT donor-to-recipient strain engraftment further underscore that the only relevant parameter is compatibility between donor and recipient, rather than the existence of a ‘gold-standard’ donor [25,26]. So far, there is insufficient and inconsistent evidence to determine the optimal protocol for using FMT in patients with CD, and thus FMT should not be used routinely in patients with CD.

## 6. Safety and Long-Term Follow-Up

### 6.1. Adverse Events and Disease Flare

No significant differences in adverse events between FMT and control were found in all UC RCTs (Cochrane RR 0.99, 95% CI 0.85–1.16). Most adverse events reported in FMT were mild or moderate and mostly self-limiting gastrointestinal symptoms [49,59]. Serious adverse events (SAEs) could not be determined because of the low number of reports; a fixed-effect meta-analysis showed a possible increase, whereas the random-effects model did not reveal an increase in SAEs [49]. The risk of IBD worsening after FMT was 14.9% across all studies, 4.6% in high-quality RCTs only, and even lower with upper-GI administration than with lower-GI administration of FMT [60]. Patients with IBD may have low rates of UC flare-ups and even more so improvements in IBD activity when treated with FMT for rCDI [61,62].

Infectious safety remains an important consideration, particularly in patients with IBD receiving immunosuppressive therapies. Rigorous donor screening, including testing for enteric pathogens and multidrug-resistant organisms, is essential to reduce the risk of infectious complications associated with FMT [63,64].

### 6.2. Long-Term Follow-Up

Long-term safety data are more extensive for patients who received FMT for rCDI than for those who received FMT for IBD. These populations should therefore not be considered interchangeable.

One of the most comprehensive long-term follow-up studies of rCDI patients was performed by Saha et al. The study included 447 patients who received FMT a median of 3.7 years before the study, and 30 were lost to follow-up. No deaths were attributed to FMT, and IBD developed in only three patients (0.7%), for an incidence rate of 0.3% per patient-year [65]. These findings did not identify a major long-term safety signal in this rCDI cohort, but the study was not designed to determine the safety of FMT in patients with established IBD or to assess risks according to immunosuppressive treatment.

The Hong Kong FMT Registry [66] also conducted a long-term follow-up study of FMT. In this study, follow-up lasted a median of 30 months. Importantly, no cases of IBD, IBS, allergy, diabetes, or psychiatric disorders developed in the study patients above the background rate for these conditions. Therefore, long-term safety data are largely available for rCDI, but IBD-specific long-term safety data are still largely lacking. Therefore, the 2024 AGA guideline recommends against using conventional FMT to treat IBD outside of clinical trials [63].

## 7. Future Directions

New trends are emerging to replace the classical FMT, which carries uncertainty, with more precise and standardizable technologies. 

While approaches like these may improve aspects of FMT such as standardization, manufacturing, and mechanisms, their long-term clinical effectiveness remains unproven. Negative or inconclusive clinical studies, after full evaluation for bias, demonstrate that having a plausible mechanism does not guarantee therapeutic benefit. The transition to a non-donor-derived FMT is an active area of research that needs more evidence; until then, controlled studies of the manufactured product, strict screening of donors or products, and long-term safety follow-up are needed. Similar statements appear in the AGA guideline regarding unresolved research questions on donor characteristics, FMT preparation, delivery, and engraftment.

### 7.1. Defined Bacterial Consortia

Rationally designed consortia aim to deliver a specific set of functions. So far, preclinical studies have shown that several defined bacterial consortia confer specific effects. A 17-strain *Clostridia* consortium induced colonic Tregs by production of SCFAs and tryptophan metabolites [1,45]. A rationally designed GUT-108 consortium was even able to reverse colitis in humanized mice [67]. An 18-strain bacterial mixture decolonized *Enterobacteriaceae* through ecological competition for nutrients [68]. Clinical studies, however, have so far been humbling. Despite their mechanistic rationale, clinical studies of SER-287, SER-301, and VE202 have not demonstrated convincing efficacy in UC, highlighting the challenges of translating preclinical findings into clinical benefit [19,69]. Early endoscopic and other biomarker-induced effects by the MH002 consortium, a six-member bacterial consortium, however, warrant further studies [70]. Defined bacterial consortia and engineered strains have evidence primarily from animal or in vitro models and need further studies.

### 7.2. Next-Generation Probiotics and Engineered Live Biotherapeutics

Engineered single strains of probiotics, such as *F. prausnitzii* strain EXL01 that is currently being investigated in clinical trial involving patients with CD [19], as well as *E. coli* Nissle secreting anti-TNFα nanobodies, as well as other Nissle derivatives that modulate ROS or IL-2, can be engineered to produce a wide array of therapeutic factors to treat IBD. Some of these strains have been made to ‘sense and respond’ to certain inflammatory mediators [18,21]. These strains are intended to deliver therapeutic factors locally in the gastrointestinal tract and may therefore provide sustained exposure to these factors at sites of inflammation. However, clinical studies have yet to establish their ability to maintain therapeutic activity over extended periods. Importantly, these strains may have the potential to prove long-term protection against relapse of IBD and thus could be investigated as maintenance therapies used to treat chronic diseases such as IBD. However, clinical trials have not yet demonstrated this potential benefit. Early human studies of selected live biotherapeutic products have provided preliminary feasibility and safety data, but convincing, replicated evidence of clinical efficacy in IBD is lacking.

### 7.3. Bacteriophage Therapy

Bacteriophages have recently emerged as an alternative to live bacteria for treating IBD. A five-phage cocktail has been shown to reduce inflammation in experimental models of colitis. Five phages have been selected against *Klebsiella pneumoniae* for treatment of IBD, and four new phage cocktails have been engineered to target AIEC (Adherent-Invasive *E. coli*) for treatment of UC [2,71]. So far, bacteriophage therapy has been proven only in experimental models.

### 7.4. Donor Selection and Combination Strategies

Moreover, the notion of a “superdonor” is being challenged. Initially, strict donor preselection based on the microbiota of patients with ulcerative colitis in the RESTORE-UC trial [72] failed to deliver sufficient improvement in patients with active UC. Recent strain-level analyses confirmed prior observations that rather the interaction between the donor and recipient microbiomes matters more than the quality of the “superdonor” itself [27]. Algorithms for matching donors and patients, preconditioning patients with antibiotics to open niches for engraftment, FMT, and combining FMT with an anti-inflammatory diet (FMT-AID) are of practical relevance in the near future [52,73]. Multi-omics approaches combined with machine-learning strategies are currently being developed to predict the FMT outcomes in individual patients based on their clinical and metagenomic data. So far, however, these approaches have not yet been validated in an independent patient cohort [74,75].

## 8. Conclusions

**Figure 1:**
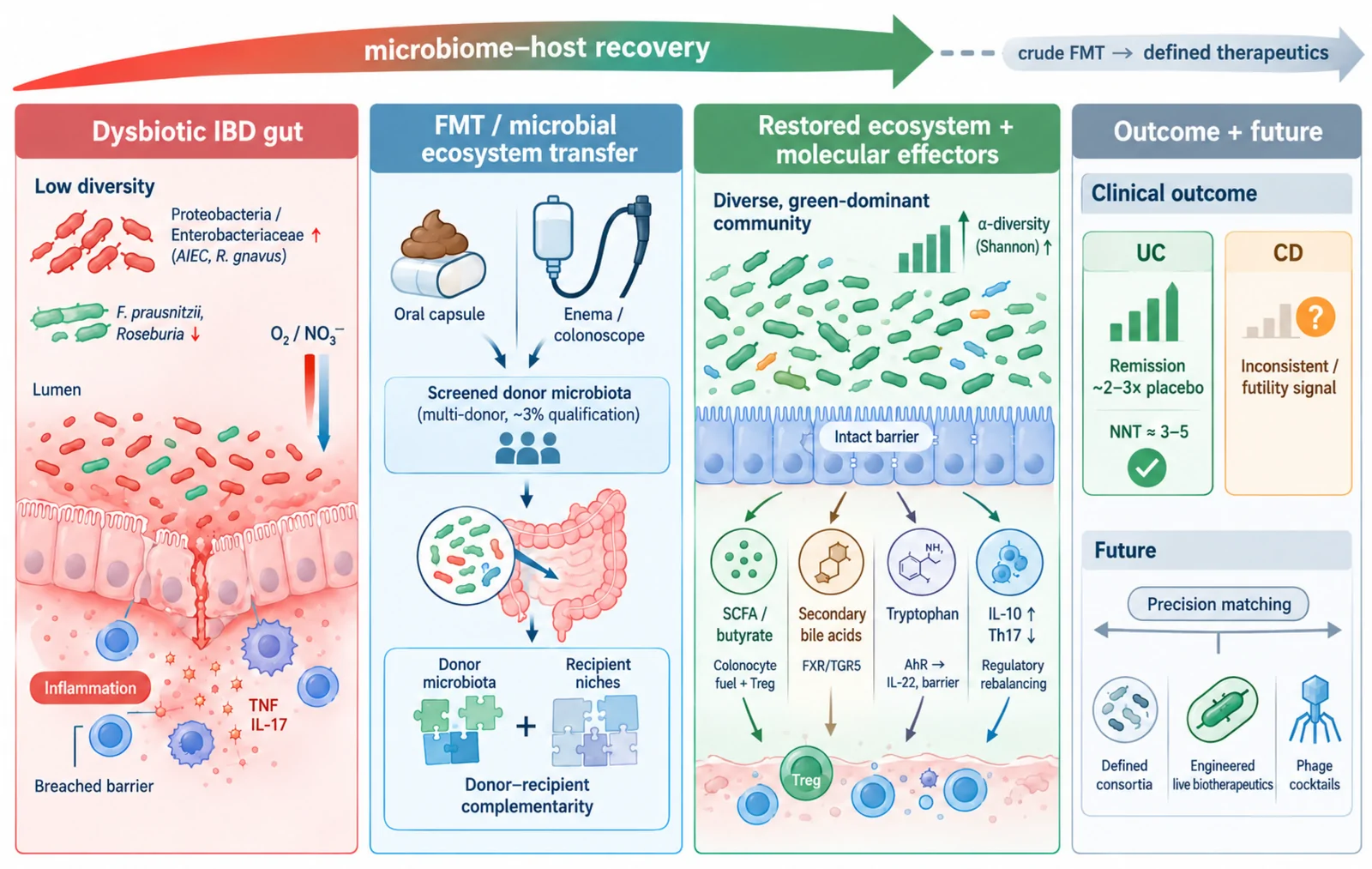
From dysbiotic inflammatory bowel disease to microbiome-based precision therapeutics.

FMT has been clinically proven to modulate gut microbiota in immune-mediated intestinal inflammation in mild-to-moderate UC. With FMT in UC, the remission rate can reach approximately double to triple the proportion of placebo-treated patients. The numbers needed to treat around 3–5 in controlled studies provide a rationale for potential application to Crohn’s disease. However, data for treatment of this condition are less consistent and even divergent, due to various reasons including ecological resistance, disease being localized to the transmural and small bowel tissue, and host genetic factors. Serious complications have occurred in rare cases; hence, the therapy currently remains under investigation for the treatment of IBD and is subject to stringent donor screening. To transform the rather crude and variable donor-dependent FMT into a defined bacterial consortium, an engineered live biotherapeutic product, or even a precision phage therapy administered to a rationally matched patient are the next steps that need to be resolved. While initial clinical trials testing these concepts have not met high expectations based on mechanistic reasoning, a clear roadmap toward defined bacterial consortia, live biotherapeutics engineered for specific indications, and precision phage therapy for specific indications has been established (Figure 1).

## 9. Literature Search Strategy

A comprehensive literature search was conducted through the PubMed database for studies published up to September 2026 to identify relevant research. The search strategy employed combinations of the following keywords: IBD, FMT, CDI, and relevant topics. Additional searches addressed clinical efficacy, mechanisms, safety, and emerging therapies. We prioritized randomized trials, systematic reviews, and meta-analyses when evaluating clinical efficacy and safety. We manually screened reference lists from relevant RCTs, systematic reviews, meta-analyses, guidelines, and major mechanistic studies. Inclusion criteria were limited to peer-reviewed articles published in English. We also manually screened the reference lists of the included articles to further confirm that the literature matched the scope of this review.

## Figures and Tables

**Table 1:** Comparative features of gut microbiota dysbiosis in ulcerative colitis and Crohn’s disease. Findings may differ according to disease activity, intestinal location, treatment exposure, sample type, sequencing platform, and bioinformatic analysis. Abbreviations: AIEC—adherent-invasive *Escherichia coli*; CD—Crohn’s disease; ESKAPE—*Enterococcus faecium*, *Staphylococcus aureus*, *Klebsiella pneumoniae*, *Acinetobacter baumannii*, *Pseudomonas aeruginosa*, and *Enterobacter* spp.; IBD—inflammatory bowel disease; SCFA—short-chain fatty acids; UC—ulcerative colitis.

Feature	Ulcerative Colitis	Crohn’s Disease	References
Overall severity of dysbiosis	Milder, heterogeneous	More severe, pronounced	[7,8]
Alpha diversity	Reduced or near normal	Markedly reduced (lowest)	[9]
Beta diversity	Heterogeneous, less separation	Clear separation from non-IBD	[7]
*Faecalibacterium prausnitzii*	Depleted (variable)	Strongly depleted	[7,8]
*Roseburia*/obligate anaerobes	Depleted	Strongly depleted	[10]
*Bacteroides*	Often enriched	Depleted	[10]
*Escherichia coli*/*Enterobacteriaceae*	Increased	Strongly increased (incl. AIEC)	[9,11]
*Ruminococcus gnavus*	Enriched	Enriched (favors CD dx)	[5,10]
ESKAPE pathogens	Not enriched	Enriched	[9]
SCFA/butyrate deficit	Modest; propionate pathway	Severe; butyrate synthesis	[12]
Secondary bile acid depletion	Present	More pronounced	[12]
Distinctive depleted taxa	*Dialister*, *Lachnospira*, *Akkermansia*, *Eubacterium rectale*	*Prevotella*, *Christensenellaceae*, *Agathobacter*, *Romboutsia*, *Butyricicoccus*	[10,11,12]

## Data Availability

No new data were created or analyzed in this study. Data sharing does not apply to this article.

## Abbreviations

The following abbreviations are used in this manuscript:

AhR	Aryl Hydrocarbon Receptor
AIEC	Adherent-Invasive *Escherichia coli*
AMP	Antimicrobial Peptide
ASCA	Anti-*Saccharomyces cerevisiae* Antibody
BA	Bile Acid
BSH	Bile Salt Hydrolase
CD	Crohn’s disease
CDAI	Crohn’s Disease Activity Index
CDEIS	Crohn’s Disease Endoscopic Index of Severity
CI	Confidence interval
DCA	deoxycholic acid
ESBL	Extended-spectrum β-lactamase
FFAR	free fatty acid receptor
FMT	Fecal microbiota transplantation
FXR	Farnesoid X Receptor
IBD	Inflammatory Bowel Disease
IL	Interleukin
IFN-γ	Interferon-gamma
JAK	Janus-kinase
LBP	Live Biotherapeutic Product
LCA	lithocholic acid
MDRO	Multidrug-resistant organism
NF-κB	nuclear factor kappa B
NNT	Number Needed to Treat
OR	Odds Ratio
rCDI	Recurrent *Clostridioides difficile* Infection
RCT	Randomized Controlled Trial
RD	Risk Difference
ROS	Reactive Oxygen Species
RR	Risk Ratio
SCFA	Short-Chain Fatty Acid
TGF-β	Transforming Growth Factor Beta
TGR5	Takeda G Protein-Coupled Bile Acid Receptor 1
Th1	T Helper Type 1 Cell
Th17	T Helper Type 17 Cell
Treg	Regulatory T cell
UC	Ulcerative Colitis
UDCA	Ursodeoxycholic Acid
ZO-1	Zonula Occludens-1

## References

[1] Chang J.T. (2020). Pathophysiology of Inflammatory Bowel Diseases. N. Engl. J. Med..

[2] Caruso R., Lo B.C., Chen G.Y., Núñez G. (2025). Host-pathobiont interactions in Crohn’s disease. Nat. Rev. Gastroenterol. Hepatol..

[3] D’Haens G.R., Jobin C. (2019). Fecal Microbial Transplantation for Diseases Beyond Recurrent Clostridium Difficile Infection. Gastroenterology.

[4] Vaughn B.P., Rank K.M., Khoruts A. (2019). Fecal Microbiota Transplantation: Current Status in Treatment of GI and Liver Disease. Clin. Gastroenterol. Hepatol..

[5] Thompson K.N., Ma S., Bhosle A., Nickols W.A., Shen J., Ghazi A.R., Dang N.H., Zhang Y., Nzabarushimana E., Kim H. (2026). Harmonized Metagenomic Signatures of the Gut Microbiome Reveal Robust Species, Functions, and Strain Links to Inflammatory Bowel Disease. Gastroenterology.

[6] Iliev I.D., Ananthakrishnan A.N., Guo C.J. (2025). Microbiota in inflammatory bowel disease: Mechanisms of disease and therapeutic opportunities. Nat. Rev. Microbiol..

[7] Caruso R., Lo B.C., Núñez G. (2020). Host-microbiota interactions in inflammatory bowel disease. Nat. Rev. Immunol..

[8] Lloyd-Price J., Arze C., Ananthakrishnan A.N., Schirmer M., Avila-Pacheco J., Poon T.W., Andrews E., Ajami N.J., Bonham K.S., Brislawn C.J. (2019). Multi-omics of the gut microbial ecosystem in inflammatory bowel diseases. Nature.

[9] Akiyama S., Nishijima S., Kojima Y., Kimura M., Ohsugi M., Ueki K., Mizokami M., Hattori M., Tsuchiya K., Uemura N. (2024). Multi-biome analysis identifies distinct gut microbial signatures and their crosstalk in ulcerative colitis and Crohn’s disease. Nat. Commun..

[10] Shah D., Phan F., Yu Z., Choi J.D.W., Toh J.W.T. (2025). Is the microbiome the answer to inflammatory bowel disease: Systematic review. Langenbecks Arch. Surg..

[11] Turpin W., Lee S.H., Croitoru K. (2025). Gut Microbiome Signature in Predisease Phase of Inflammatory Bowel Disease: Prediction to Pathogenesis to Prevention. Gastroenterology.

[12] Wang Z., Shi J., Yu Y., Zhu L., Zhu L., Tuo W., Ni J., Wu Y., Qiu S., Yu Y. (2026). Gut microbiota and bile acids profiles study of ulcerative colitis and Crohn’s disease patients. Front. Microbiol..

[13] Segal J.P., Mullish B.H., Quraishi M.N., Iqbal T., Marchesi J.R., Sokol H. (2020). Mechanisms underpinning the efficacy of faecal microbiota transplantation in treating gastrointestinal disease. Ther. Adv. Gastroenterol..

[14] Cardenas L.A.C., Leonard M.M., Baldridge M.T., Handley S.A. (2026). Gut virome dynamics: From commensal to critical player in health and disease. Nat. Rev. Gastroenterol. Hepatol..

[15] Lam S., Bai X., Shkoporov A.N., Park H., Wu X., Lan P., Zuo T. (2022). Roles of the gut virome and mycobiome in faecal microbiota transplantation. Lancet Gastroenterol. Hepatol..

[16] Zhao X., Hu X., Han J., Yin R., Zhang S., Liu H. (2023). Gut mycobiome: A “black box” of gut microbiome-host interactions. WIREs Mech. Dis..

[17] Lavelle A., Sokol H. (2020). Gut microbiota-derived metabolites as key actors in inflammatory bowel disease. Nat. Rev. Gastroenterol. Hepatol..

[18] Zhu Q., Feng S., Yan Z., Wang Z., Huang X., Sun C., Zhang B., Li X., Gu Y. (2026). Engineered *Escherichia coli* Nissle 1917 secreting anti-TNF-α nanobody as a single-strain live biotherapeutic for inflammatory bowel disease. Front. Immunol..

[19] Bethlehem L., Estevinho M.M., Grinspan A., Magro F., Faith J.J., Colombel J.F. (2024). Microbiota therapeutics for inflammatory bowel disease: The way forward. Lancet Gastroenterol. Hepatol..

[20] DuPont H.L., Jiang Z.D., DuPont A.W., Utay N.S. (2020). Abnormal Intestinal Microbiome in Medical Disorders and Potential Reversibility by Fecal Microbiota Transplantation. Dig. Dis. Sci..

[21] Srivastava R., Lesser C.F. (2024). Living Engineered Bacterial Therapeutics: Emerging Affordable Precision Interventions. Microb. Biotechnol..

[22] Bénard M.V., de Goffau M.C., Blonk J., Hugenholtz F., van Buuren J., Paramsothy S., Kaakoush N.O., D’Haens G.R.A.M., Borody T.J., Kamm M.A. (2025). Gut Microbiota Features in Relation to Fecal Microbiota Transplantation Outcome in Ulcerative Colitis: A Systematic Review and Meta-Analysis. Clin. Gastroenterol. Hepatol..

[23] Rossen N.G., Fuentes S., van der Spek M.J., Tijssen J.G., Hartman J.H.A., Duflou A., Löwenberg M., van den Brink G.R., Mathus-Vliegen E.M.H., de Vos W.M. (2015). Findings From a Randomized Controlled Trial of Fecal Transplantation for Patients with Ulcerative Colitis. Gastroenterology.

[24] Paramsothy S., Kamm M.A., Kaakoush N.O., Walsh A.J., van den Bogaerde J., Samuel D., Leong R.W.L., Connor S., Ng W., Paramsothy R. (2017). Multidonor intensive faecal microbiota transplantation for active ulcerative colitis: A randomised placebo-controlled trial. Lancet.

[25] Kong L., Lloyd-Price J., Vatanen T., Seksik P., Beaugerie L., Simon T., Vlamakis H., Sokol H., Xavier R.J. (2020). Linking Strain Engraftment in Fecal Microbiota Transplantation with Maintenance of Remission in Crohn’s Disease. Gastroenterology.

[26] Ianiro G., Punčochář M., Karcher N., Porcari S., Armanini F., Asnicar F., Beghini F., Blanco-Míguez A., Cumbo F., Manghi P. (2022). Variability of strain engraftment and predictability of microbiome composition after fecal microbiota transplantation across different diseases. Nat. Med..

[27] Schmidt T.S.B., Li S.S., Maistrenko O.M., Akanni W., Coelho L.P., Dolai S., Fullam A., Glazek A.M., Hercog R., Herrema H. (2022). Drivers and determinants of strain dynamics following fecal microbiota transplanta-tion. Nat. Med..

[28] Zhang B., Yang L., Ning H., Cao M., Chen Z., Chen Q., Lian G., Tang H., Wang Q., Wang J. (2023). A Matching Strategy To Guide Donor Selection for Ulcerative Colitis in Fecal Microbiota Transplantation: Meta-Analysis and Analytic Hierarchy Process. Microbiol. Spectr..

[29] Paramsothy S., Nielsen S., Kamm M.A., Deshpande N.P., Faith J.J., Clemente J.C., Paramsothy R., Walsh A.J., van den Bogaerde J., Samuel D. (2019). Specific Bacteria and Metabolites Associated with Response to Fecal Microbiota Transplantation in Patients with Ulcerative Colitis. Gastroenterology.

[30] Mann E.R., Lam Y.K., Uhlig H.H. (2024). Short-chain fatty acids: Linking diet, the microbiome and immunity. Nat. Rev. Immunol..

[31] Salaga M., Bartoszek A., Binienda A., Krajewska J.B., Fabisiak A., Mosińska P., Dziedziczak K., Niewinna K., Talar M., Tarasiuk A. (2021). Activation of Free Fatty Acid Receptor 4 Affects Intestinal Inflammation and Improves Colon Permeability in Mice. Nutrients.

[32] Spathakis M., Dovrolis N., Filidou E., Kandilogiannakis L., Tarapatzi G., Valatas V., Drygiannakis I., Paspaliaris V., Arvanitidis K., Manolopoulos V.G. (2024). Exploring Microbial Metabolite Receptors in Inflammatory Bowel Disease: An In Silico Analysis of Their Potential Role in Inflammation and Fibrosis. Pharmaceuticals.

[33] Collins S.L., Stine J.G., Bisanz J.E., Okafor C.D., Patterson A.D. (2023). Bile acids and the gut microbiota: Metabolic interactions and impacts on disease. Nat. Rev. Microbiol..

[34] Lee M.H., Nuccio S.-P., Mohanty I., Hagey L.R., Dorrestein P.C., Chu H., Raffatellu M. (2024). How bile acids and the microbiota interact to shape host immunity. Nat. Rev. Immunol..

[35] Lu G., Zhang S., Wang R., Wu X., Chen Y., Wen Q., Cui B., Zhang F., Li P. (2025). Fecal microbiota transplantation improves bile acid malabsorption in patients with inflammatory bowel disease: Results of microbiota and metabolites from two cohort studies. BMC Med..

[36] Geng S., Cheng S., Li Y., Wen Z., Ma X., Jiang X., Wang Y., Han X. (2018). Faecal Microbiota Transplantation Reduces Suscepti-bility to Epithelial Injury and Modulates Tryptophan Metabolism of the Microbial Community in a Piglet Model. J. Crohn’s Colitis.

[37] Michaudel C., Danne C., Agus A., Magniez A., Aucouturier A., Spatz M., Lefevre A., Kirchgesner J., Rolhion N., Wang Y. (2023). Rewiring the altered tryp-tophan metabolism as a novel therapeutic strategy in inflammatory bowel diseases. Gut.

[38] Burrello C., Garavaglia F., Cribiù F.M., Ercoli G., Lopez G., Troisi J., Colucci A., Guglietta S., Carloni S., Guglielmetti S. (2018). Therapeutic faecal microbiota transplantation con-trols intestinal inflammation through IL10 secretion by immune cells. Nat. Commun..

[39] Luu L.D.W., Pandey A., Paramsothy S., Ngo C., Castaño-Rodríguez N., Liu C., Kamm M.A., Borody T.J., Man S.M., Kaakoush N.O. (2024). Profiling the colonic mucosal response to fecal microbiota transplantation identifies a role for GBP5 in colitis in humans and mice. Nat. Commun..

[40] Danne C., Rolhion N., Sokol H. (2021). Recipient factors in faecal microbiota transplantation: One stool does not fit all. Nat. Rev. Gastroenterol. Hepatol..

[41] Martín R., Rios-Covian D., Huillet E., Auger S., Khazaal S., Bermúdez-Humarán L.G., Sokol H., Chatel J.M., Langella P. (2023). Faecalibacterium: A bacterial genus with promising human health applications. FEMS Microbiol. Rev..

[42] Majzoub M.E., Paramsothy S., Haifer C., Parthasarathy R., Borody T.J., Leong R.W., Kamm M.A., Kaakoush N.O. (2024). The phageome of patients with ulcerative colitis treated with donor fecal microbiota reveals markers associated with disease re-mission. Nat. Commun..

[43] Leonardi I., Paramsothy S., Doron I., Semon A., Kaakoush N.O., Clemente J.C., Faith J.J., Borody T.J., Mitchell H.M., Colombel J.F. (2020). Fungal Trans-kingdom Dynamics Linked to Responsiveness to Fecal Microbiota Transplantation (FMT) Therapy in Ulcerative Colitis. Cell Host Microbe.

[44] Quraishi M.N., Moakes C.A., Yalchin M., Blackwell C., Segal J., Ives N.J., Magill L., Manzoor S.E., Gerasimidis K., McMullan C. (2026). Mechanistic insights into fecal microbiota transplantation for the treatment of ulcerative colitis: Analysis of the STOP-Colitis trial. J. Crohn’s Colitis.

[45] Oka A., Bongers G., Mishima Y., Baltus A.J., Gray S.M., Liu B., Herzog J.W., Benedetto J.R., Fan T.J., Jang J. (2026). Defined human Clostridia consortia reverse colitis via dual effects of tryptophan metabolites on microbiota and immunity. Cell Host Microbe.

[46] Costello S.P., Hughes P.A., Waters O., Bryant R.V., Vincent A.D., Blatchford P., Katsikeros R., Makanyanga J., Cam-paniello M.A., Mavrangelos C. (2019). Effect of Fecal Microbiota Transplantation on 8-Week Remission in Patients with Ulcerative Colitis: A Randomized Clinical Trial. JAMA.

[47] Haifer C., Paramsothy S., Kaakoush N.O., Saikal A., Ghaly S., Yang T., Luu L.D.W., Borody T.J., Leong R.W. (2022). Lyophilised oral faecal microbiota transplantation for ulcerative colitis (LOTUS): A randomised, double-blind, placebo-controlled trial. Lancet Gastroenterol. Hepatol..

[48] Moayyedi P., Surette M.G., Kim P.T., Libertucci J., Wolfe M., Onischi C., Armstrong D., Marshall J.K., Kassam Z., Reinisch W. (2015). Fecal Microbiota Transplantation Induces Remission in Patients with Active Ulcerative Colitis in a Randomized Controlled Trial. Gastroenterology.

[49] Imdad A., Pandit N.G., Zaman M., Minkoff N.Z., Tanner-Smith E.E., Gomez-Duarte O.G., Acra S.A., Nicholson M.R. (2023). Fecal transplantation for treatment of inflammatory bowel disease. Cochrane Database Syst. Rev..

[50] Narula N., Kassam Z., Yuan Y., Colombel J.F., Ponsioen C., Reinisch W., Moayyedi P. (2017). Systematic Review and Meta-analysis: Fecal Microbiota Transplantation for Treatment of Active Ulcerative Colitis. Inflamm. Bowel Dis..

[51] El Hage Chehade N., Ghoneim S., Shah S., Chahine A., Mourad F.H., Francis F.F., Binion D.G., Farraye F.A., Hashash J.G. (2023). Efficacy of Fecal Microbiota Transplantation in the Treatment of Active Ulcerative Colitis: A Systematic Review and Me-ta-Analysis of Double-Blind Randomized Controlled Trials. Inflamm. Bowel Dis..

[52] Huang B., An H., Qiu Y., Ye T., Huang Y., Zheng M., Shi D., Su Y., Wang R. (2026). Comparative Effectiveness and Safety of Fecal Microbiota Transplantation in Ulcerative Colitis: An Updated Systematic Review and Meta-Analysis. Adv. Ther..

[53] Sood A., Mahajan R., Singh A., Midha V., Mehta V., Narang V., Singh T., Pannu A.S. (2019). Role of Faecal Microbiota Transplantation for Maintenance of Remission in Patients with Ulcerative Colitis: A Pilot Study. J. Crohns Colitis..

[54] Kedia S., Virmani S., K Vuyyuru S., Kumar P., Kante B., Sahu P., Kaushal K., Farooqui M., Singh M., Verma M. (2022). Faecal microbiota transplantation with anti-inflammatory diet (FMT-AID) followed by anti-inflammatory diet alone is effective in inducing and maintaining remission over 1 year in mild to moderate ulcerative colitis: A randomised controlled trial. Gut.

[55] Sokol H., Landman C., Seksik P., Berard L., Montil M., Nion-Larmurier I., Bourrier A., Le Gall G., Lalande V., De Rougemont A. (2020). Fecal microbiota transplantation to maintain remission in Crohn’s disease: A pilot randomized controlled study. Microbiome.

[56] Kao D., Wong K., Jijon H., Moayyedi P., Franz R., McDougall C., Hotte N., Panaccione R., Semlacher E., Kroeker K.I. (2024). Preliminary Results From a Mul-ticenter, Randomized Trial Using Fecal Microbiota Transplantation to Induce Remission in Patients with Mild-to-Moderate Crohn’s Disease. Am. J. Gastroenterol..

[57] Yang Z., Bu C., Yuan W., Shen Z., Quan Y., Wu S., Zhu C., Wang X. (2020). Fecal Microbiota Transplant via Endoscopic Delivering Through Small Intestine and Colon: No Difference for Crohn’s Disease. Dig. Dis. Sci..

[58] Zhou S., Cui Y., Zhang Y., Zhao T., Cong J. (2023). Fecal microbiota transplantation for induction of remission in Crohn’s disease: A systematic review and meta-analysis. Int. J. Colorectal Dis..

[59] Feng J., Chen Y., Liu Y., Lin L., Lin X., Gong W., Xia R., He J., Sheng J., Cai H. (2023). Efficacy and safety of fecal microbiota transplantation in the treatment of ulcerative colitis: A systematic review and meta-analysis. Sci. Rep..

[60] Qazi T., Amaratunga T., Barnes E.L., Fischer M., Kassam Z., Allegretti J.R. (2017). The risk of inflammatory bowel disease flares after fecal microbiota transplantation: Systematic review and meta-analysis. Gut Microbes.

[61] Allegretti J.R., Kelly C.R., Grinspan A., Mullish B.H., Hurtado J., Carrellas M., Marcus J., Marchesi J.R., McDonald J.A.K., Gerardin Y. (2021). Inflammatory Bowel Disease Outcomes Following Fecal Microbiota Transplantation for Recurrent C. difficile Infection. Inflamm. Bowel Dis..

[62] van Lingen E.E., Baunwall S.S.M.D., Lieberknecht S.S.C., Benech N.N., Ianiro G.G., Sokol H.H., Gasbarrini A.A., Cam-marota G.G., Eriksen M.M.K., van der Meulen-de Jong A.A.E. (2023). Short- and long-term follow-up after fecal microbiota transplantation as treatment for recurrent Clostridioides difficile infection in patients with inflammatory bowel disease. Ther. Adv. Gastroenterol..

[63] Peery A.F., Kelly C.R., Kao D., Vaughn B.P., Lebwohl B., Singh S., Imdad A., Altayar O. (2024). AGA Clinical Practice Guideline on Fecal Microbiota-Based Therapies for Select Gastrointestinal Diseases. Gastroenterology.

[64] Lai C.Y., Sung J., Cheng F., Tang W., Wong S.H., Chan P.K.S., Kamm M.A., Sung J.J.Y., Kaplan G., Chan F.K.L. (2019). Systematic review with meta-analysis: Review of donor features, procedures and outcomes in 168 clinical studies of faecal microbiota transplantation. Aliment. Pharmacol. Ther..

[65] Saha S., Mara K., Pardi D.S., Khanna S. (2021). Long-term Safety of Fecal Microbiota Transplantation for Recurrent Clostridioides difficile Infection. Gastroenterology.

[66] Yau Y.K., Lau L.H.S., Lui R.N.S., Wong S.H., Guo C.L., Mak J.W.Y., Ching J.Y.L., Ip M., Kamm M.A., Rubin D.T. (2024). Long-Term Safety Outcomes of Fecal Microbiota Transplantation: Real-World Data Over 8 Years From the Hong Kong FMT Registry. Clin. Gastroenterol. Hepatol..

[67] van der Lelie D., Oka A., Taghavi S., Umeno J., Fan T.J., Merrell K.E., Watson S.D., Ouellette L., Liu B., Awoniyi M. (2021). Rationally designed bacterial consortia to treat chronic immune-mediated colitis and restore intestinal homeostasis. Nat. Commun..

[68] Furuichi M., Kawaguchi T., Pust M.-M., Yasuma-Mitobe K., Plichta D.R., Hasegawa N., Ohya T., Bhattarai S.K., Sasajima S., Aoto Y. (2024). Commensal consortia decolonize Enterobacteriaceae via ecological control. Nature.

[69] Newman K.L., Allegretti J.R. (2024). Emerging Noninfectious Indications for Live Biotherapeutic Products in Gastroenterology. Am. J. Gastroenterol..

[70] Guarino-Vignon P., Louis E., Pham H.-P., Orianne G., Tkacz E., Brot L., Mazzetti E., Sedda D., Ruffié P., Rolhion N. (2026). Faecalibacterium prausnitzii EXL01 for the Maintenance of Steroid-induced Clinical Response or Remission in Patients with Crohn’s Disease: A first in human trial. Nat. Commun..

[71] Federici S., Kredo-Russo S., Valdés-Mas R., Kviatcovsky D., Weinstock E., Matiuhin Y., Silberberg Y., Atarashi K., Furuichi M., Oka A. (2022). Targeted suppression of human IBD-associated gut microbiota commensals by phage consortia for treatment of intestinal inflammation. Cell.

[72] Caenepeel C., Deleu S., Castellanos J.F.V., Arnauts K., Braekeleire S., Machiels K., Baert F., Mana F., Pouillon L., Hindryckx P. (2025). Rigorous Donor Selection for Fecal Microbiota Transplantation in Active Ulcerative Colitis: Key Lessons From a Randomized Controlled Trial Halted for Futility. Clin. Gastroenterol. Hepatol..

[73] Zhang Y.J., Bousvaros A., Docktor M., Kaplan A.L., Rufo P.A., Leier M., Weatherly M., Zimmerman L., Nguyen L.T.T., Barton B. (2024). Higher alpha diversity and Lactobacillus blooms are associated with better en-graftment after fecal microbiota transplant in inflammatory bowel disease. Sci. Rep..

[74] Mao R., Chen M. (2022). Precision medicine in IBD: Genes, drugs, bugs and omics. Nat. Rev. Gastroenterol. Hepatol..

[75] Rozera T., Pasolli E., Segata N., Ianiro G. (2025). Machine Learning and Artificial Intelligence in the Multi-Omics Approach to Gut Microbiota. Gastroenterology.

